# Do Mitochondria Limit Hot Fish Hearts? Understanding the Role of Mitochondrial Function with Heat Stress in *Notolabrus celidotus*

**DOI:** 10.1371/journal.pone.0064120

**Published:** 2013-05-28

**Authors:** Fathima I. Iftikar, Anthony J. R. Hickey

**Affiliations:** School of Biological Sciences, University of Auckland, Auckland, New Zealand; Instituto Nacional de Cardiologia, Mexico

## Abstract

Hearts are the first organs to fail in animals exposed to heat stress. Predictions of climate change mediated increases in ocean temperatures suggest that the ectothermic heart may place tight constraints on the diversity and distribution of marine species with cardiovascular systems. For many such species, their upper temperature limits (T_max_) and respective heart failure (HF) temperature (T_HF_) are only a few degrees from current environmental temperatures. While the ectothermic cardiovascular system acts as an “ecological thermometer,” the exact mechanism that mediates HF remains unresolved. We propose that heat-stressed cardiac mitochondria drive HF. Using a common New Zealand fish, *Notolabrus celidotus*, we determined the T_HF_ (27.5°C). Haemoglobin oxygen saturation appeared to be unaltered in the blood surrounding and within heat stressed hearts. Using high resolution respirometry coupled to fluorimeters, we explored temperature-mediated changes in respiration, ROS and ATP production, and overlaid these changes with T_HF_. Even at saturating oxygen levels several mitochondrial components were compromised before T_HF_. Importantly, the capacity to efficiently produce ATP in the heart is limited at 25°C, and this is prior to the acute T_HF_ for *N. celidotus*. Membrane leakiness increased significantly at 25°C, as did cytochrome *c* release and permeability to NADH. Maximal flux rates and the capacity for the electron transport system to uncouple were also altered at 25°C. These data indicate that mitochondrial membrane integrity is lost, depressing ATP synthesis capacity and promoting cytochrome *c* release, prior to T_HF._ Mitochondria can mediate HF in heat stressed hearts in fish and play a significant role in thermal stress tolerance, and perhaps limit species distributions by contributing to HF.

## Introduction

Increase in ocean temperatures globally, present concerns for ectotherms that live in these oceans as they are generally sensitive to fluctuating temperatures [1]–[3]. By definition, body temperatures of ectotherms change with their environment. As a consequence the capacity to adapt to environmental temperature fluctuations is dependent on altering biochemical and metabolic processes in order to maintain homeostasis [2], [4]–[9]. Metabolic rates of ectotherms typically increase with acute rises in habitat temperature and this elevates demands on precious metabolic fuels [10]. If metabolic rates increase drastically or if they run inefficiently, fuel reserves must be redirected from anabolic processes to power catabolism. This will impair growth, physiological equilibrium, and ultimately survival. Therefore, heat stress has complex and integrative effects on circulation, respiration, digestion, growth, reproduction, and locomotive capacities of ectotherms [11].

For ectotherms with cardiovascular systems the heart is temperature sensitive, and in most cases the critical temperature for heart failure (T_HF_) is only a few degrees above the upper habitat temperatures (T_max_) [8], [11], [12]. For marine ectotherms these apparently fine margins between T_max_ and T_HF_ may have restructured species distributions. With respect to fish, the main causative limitation is believed to be cardiac function. Acute heart failure (HF) in ectotherms has been proposed to result from decreased oxygen availability [12], [13], because rising habitat temperatures decreases blood oxygen solubility while metabolic rates increase. However, oxygen diffusion rates are enhanced at elevated temperatures and to a limit these can offset decreases in oxygen solubility [14], [15]. Although oxygen solubility ultimately does become limiting, this condition occurs above temperatures experienced by most tropical organisms where coincidentally there is the greatest species diversity [15]. Additionally, increasing temperature can further limit ectotherm cardiac function physiologically since temperature affects the cardiac pacemaker directly disrupting both signal production and transduction [16]. It has also been suggested that hearts fail with elevated temperature because calcium handling rates become inadequate in cardiac myocytes during excitation-contraction coupling [17]. However, calcium dynamics appear to be maintained across broad temperature ranges despite acute temperature effects on individual proteins [18]. Therefore, if oxygen solubility and calcium handling are not limiting cardiac function at high temperatures, what other mechanisms could explain the T_HF_?

Another line of evidence implicate mitochondria as instigators of HF. Mitochondria are central to HF in numerous cardiac diseases [19]–[21], and as temperature approaches T_max_, succinate (a tricarboxylic acid (*TCA*) cycle intermediate) has been reported to increase in concentration in the blood of heat stressed fish [12]. Succinate is a mitochondrial electron transport system (ETS) substrate that feeds electrons into complex II and the plasma membrane is normally impermeable to this metabolite [22], [23]. For vertebrates the appearance of succinate in blood indicates mitochondrial dysfunction [24]. Mitochondria occupy 20 to 40% of the vertebrate cardiomyocyte volume and channel 90% of the energy as ATP to contractile machinery and ion pumps [25]. The proteomes [26], structures, dynamics [27], and energetic outputs [28] make heart mitochondria different to mitochondria from other tissues as they are sensitive to heat stress, ischemic damage, and oxidative stress [19], [29]–[31].

Cardiac mitochondria have been explored in contexts of HF in mammals exposed to heat stress [20], [32]–[35]. The respiratory control ratio (RCR) and the phosphate/oxygen (P:O) ratio are measures of mitochondrial uncoupling and putatively oxidative phosphorylation efficiency, respectively. These ratios decreased in isolated mitochondria from cardiomyocytes of rats that were thermally challenged [36]. These alterations to cardiac mitochondria should depress cellular ATP thereby driving necrosis. Thermally challenged heart mitochondria can also elevate reactive oxygen species (ROS) release and promote apoptosis [27], [29], [37]. Both necrosis and apoptosis can contribute to HF [29]. There are no studies to date that examine if cardiac mitochondria contribute to thermally induced HF in ectotherms such as fish.

**Figure 1:**
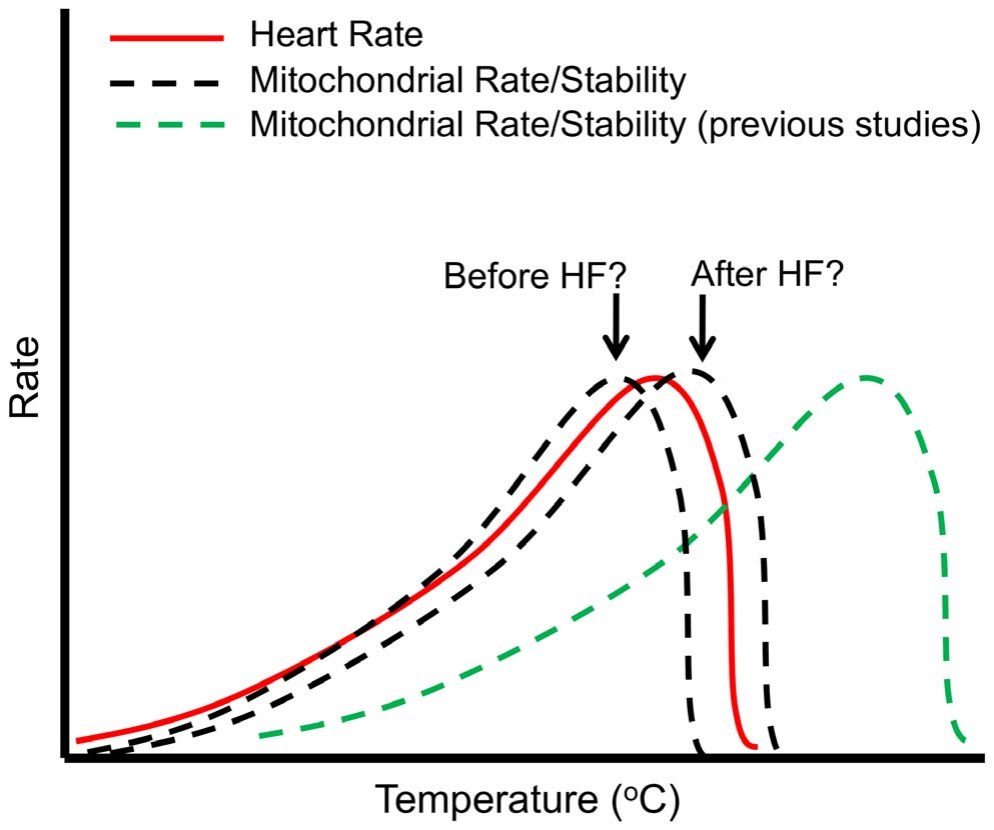
Understanding thermal limits of HF. Previous studies showed that mitochondria were robust beyond temperatures at which the heart fails (green dashed line). This study questions whether cardiac mitochondria fail before (a causal mechanism) or after heart failure (an effect, red line)?

The majority of studies investigating temperature influences on ectotherm mitochondria have reported stable respiration rates at temperatures equal to or well above species T_max_ [8], [11]. Therefore, the role of heart mitochondria in thermally-induced HF has been discounted (figure 1). In general, these studies have not measured the efficiency or stability of mitochondria. Most studies used substrates that did not fully test respirational flux [38], [39], and sometimes with non-physiologically relevant buffers [40], [41]. Some of these studies compared disparate species [42], [43] or species from extreme thermal environments [44], [45]. Significantly, few have investigated the pivotal role of heart mitochondria in a common species from temperate marine environments [46], [47].

This study determined the effects of increasing temperature on cardiac mitochondria from a common and abundant New Zealand marine fish, *Notolabrus celidotus* [48]. This species is an appropriate model organism as it is a food source for a number of finfish and therefore, provides insight into the effects that increasing ocean temperatures could present at species and ecosystem levels. The primary aim was to test whether mitochondrial function is potentially a cause or an effect of HF (figure 1). We first determined the T_HF_ of *N. celidotus* and assessed cardiac mitochondrial function from heat stressed fish following HF, compared to non heat stressed fish (controls). Mitochondrial function was examined using saponin permeabilized cardiac fibres. Permeabilization leaves cardiac mitochondria and their cytoskeleton contacts intact and provide a more physiologically relevant preparation in terms of ROS production and stability [49]. We then tested oxygen fluxes through different components of the ETS and oxidative phosphorylation (OXP) systems to identify the most susceptible points following acute heat stress. Lastly, we evaluated the affinity of heart mitochondria for pyruvate, ROS production and the capacity to synthesize ATP in maximal respiration states to understand the mechanistic contributions of mitochondria to HF.

## Materials and Methods

### Experimental Animals

Fish were caught using hand-held line fishing from piers around the greater Auckland region. No specific permits were required for this method of acquiring fish because areas frequented were available for public recreational fishing. No permits were required for number of fish caught since *N. celidotus* is not a protected or endangered species. They were kept at 18±0.5°C in aerated aquaria with recirculating seawater under a 12 hour light photoperiod for 4 weeks prior to experiments. Fish were fed every two days with green lipped mussel and feeding was suspended 48 hours prior to experiments. All experiments and procedures met with the ethical requirements and recommendations of the Animal Ethics Committee of the University of Auckland, New Zealand (Permit approval AEC/04/2009/R720 Fish).

### Measuring Cardiac Function

Following a similar protocol described previously [47], fish heart rates were measured using foetal Doppler probes (Sonotrax B, Contact Medical Systems, China) without anaesthesia as anaesthetics can affect mitochondrial function [50]. Fish were secured ventral side up within a submerged sponge holder in a 4 L plastic container that was then immersed in a larger 20 L water reservoir creating a recirculating system with constantly aerated seawater (18.0±0.5°C). Seawater was pumped across the gills using a small pump (Rio®, mini 150, Taipei, Taiwan) at a rate of 40±0.1 mL. min^−1^ into the buccal cavity to induce atonic immobility [51]. A damp black cloth with a 2 cm diameter opening was placed over the fish just behind the gills exposing the underside for placement of the Doppler probe. Fish were held prone for 3 hours prior to experiments to ensure the heart rate had settled and was constant. A thermocouple (Digitech QM-1600) was then placed inside the fish’s mouth to record temperature that was gradually increased 1°C every 10 minutes in the 20 L reservoir tank using glass aquarium heaters [52]–[54].

Sonograms were measured over one minute immediately after each temperature was reached (*N* = 8, mean mass 35.93±3.44 g). Previous trials found that the fish heartbeat became inconsistent or intermittent just prior to death. The experiment was terminated when the heartbeat became inconsistent and this temperature represented T_HF_. Control fish (*N* = 8, mean mass 25.88±1.88 g) were maintained at 18±0.5°C, and sonograms were measured every 30 minutes for the duration of the temperature-exposure experiments. The Doppler audio output was connected to a PC soundcard via an audio jack and recorded using Audacity® 1.2.6 (http://audacity.sourceforge.net/). Fish were then euthanized by concussion and a heparinized caudal blood sample was taken. Plasma was separated by centrifugation (5 minutes at 2500 rcf), frozen in liquid nitrogen and stored at −80°C for metabolomic analysis. The heart was excised for mitochondrial respirometry described below.

### Assessing Blood Oxygen Saturation

Haemoglobin saturation was monitored across the heart non-invasively using spectroscopy. As oxygenated haemoglobin absorbs at near infrared wavelengths around 940 nm and deoxygenated haemoglobin absorbs at 600 nm [55], a 3 Watt infrared LED (peak wavelength 940 nm) and a 3 Watt red light-emitting LED (peak wavelength 600 nm) were placed on one side of the fish near the base of the pectoral fins. The transmitted light through the fish heart was received on the opposite side by a 1.5 mm Perspex fibre optic cable attached to an Ocean Optics USB4000 spectrometer. Data were acquired using Ocean Optics SpectraSuite software. As haemoglobin de-saturates the absorption peak wavelength at 600 nm increases, whereas the absorption at 940 nm decreases [55]. Preliminary experiments showed that the 940∶600 ratio decreased with haemoglobin desaturation on exposure of fish to brief hypoxia (N_2_ exposure). As above, experimental fish (*N* = 6, mean mass 117.75±22.24 g) were exposed to increasing temperatures (1°C every 10 minutes) until T_HF_, while control fish (*N* = 4, mean mass 92.0±13.80 g) were maintained at 18±0.5°C and measured over the same time duration as experimental fish.

### Plasma Metabolic Profile

Plasma metabolites were extracted using −30°C methanol according to a modified protocol [56]. Initially 20 µL of internal standard (10 mM solution of 2,3,3,3-d4 DL-Alanine) was added to 100 µL of plasma, vortexed and frozen at −80°C. Plasma samples were freeze dried (Virtis freeze dryer) and the metabolites extracted by adding 500 µL methanol:water (1∶1 v/v) at −30°C. The solution was vortexed vigorously for one minute and then centrifuged at 4°C for five minutes at 16,000* g.* Supernatants were collected and centrifuged again. The pellets were re-suspended and extracted a second time in 500 µL −30°C methanol:water (4∶1 v/v) and pooled with the first extract. Extracted metabolites were freeze dried following the addition of 5 mL of cold bi-distilled water (4°C). Metabolites were chemically derivatized using methyl chloroformate and the samples analyzed by gas chromatography-mass spectrometry (GC-MS) [56].

### Tissue Metabolites and Enzyme Markers

Lactate, citrate synthase (CS, an aerobic marker of mitochondrial content [57], [58]) and lactate dehydrogenase (LDH, an anaerobic marker enzyme [59]) in cardiac tissue from control and heat stressed fish were measured similar to Iftikar et al. [47] with modifications from previous studies [60], [61]. Glucose-6-phosphate dehydrogenase (G6PDH), a key enzyme in the pentose phosphate pathway, was analyzed according to McClelland et al. [62]. All assays were measured at 25°C.

### Mitochondrial Bioenergetics

#### Fibre preparation for mitochondrial respirometry

In all mitochondrial assays the preparation of heart fibres followed the same protocol. Fish hearts were rapidly dissected and then immersed in 2 mL modified cold relaxing buffer (BIOPS: 2.77 mM CaK_2_EGTA, 7.23 mM K_2_EGTA, 5.77 mM Na_2_ATP, 6.56 mM MgCl_2_·6 H_2_O, 20 mM taurine, 20 mM imidazole, 0.5 mM dithiothreitol, 50 mM K-MES, 15 mM Na-phosphocreatine and 50 mM Sucrose, pH 7.1 at 0 °C). The dissected heart was teased into fibre bundles using a dissecting microscope and placed in 1 mL cold BIOPS (4°C) in a plastic culture plate. Fibres were then transferred to fresh BIOPS containing 50 µg. mL^−1^ saponin in a 12 well-culture plate, and gently shaken on ice for 30 minutes. Consequently, fibres were transferred and washed three times for 10 minutes in 2 mL of modified mitochondrial respiratory medium (Fish-MiRO5∶0.5 mM EGTA, 3 mM MgCl_2_·6 H_2_O, 60 mM K-lactobionate, 20 mM taurine, 10 mM KH_2_PO_4_, 20 mM HEPES, 160 mM sucrose and 1 g. L^−1^ BSA, essentially free fatty acid, pH 7.24 at 20°C). Fibres were blotted dry on filter paper and weighed into 2–3 mg bundles for respiration assays. All chemicals were obtained from Sigma-Aldrich (St. Louis, MO, USA).

#### Testing mitochondrial function in permeabilized cardiac fibres

Four experiments were conducted to assess heart mitochondrial function. The first two experiments applied identical protocols that measured respiration and ROS production simultaneously. However, the first tested the effect of acute heat stress on hearts *in vivo* and assayed fibres at 20°C. The second tested mitochondrial function across a range of temperatures using fibres from naive fish. The third experiment tested apparent affinities of fibres for substrates across a range of temperatures, and the last measured ATP synthesis across a range of temperatures.

#### Experiment 1: Testing the effects of acute temperature exposure *in vivo*

**Figure 2:**
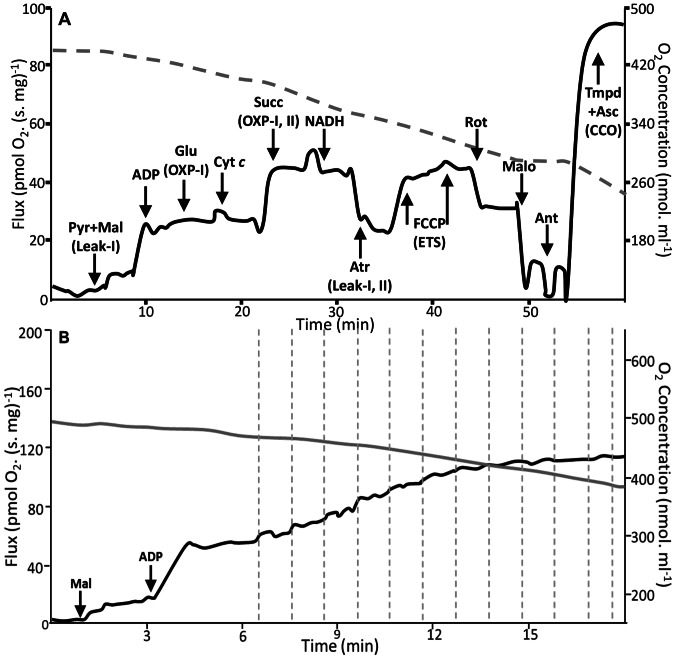
Representative mitochondrial respiration assay traces from permeabilized cardiac fibres measuring (A) mitochondrial flux (pmol O_2_. s^−1^. mg^−1^, black line, left y-axis) and oxygen concentration (nmol. ml^− 1^, dotted grey line, right y-axis) over time (mins). Titrations of mitochondrial substrates, poisons and inhibitors and their time of addition are shown with arrows, and the resulting *respiratory state* in parenthesis. **Pyr** [pyruvate], **Mal** [malate], **Glu** [glutamate], **Cyt** ***c*** [cytochrome-*c*], **Succ** [succinate], **Atr** [atractyloside], **FCCP** [carbonyl cyanide p-(trifluoromethoxy) phenyl-hydrazone], **Rot** [rotenone], **Malo** [malonate], **Ant** [antimycin-a], **TMPD** [N, N, N’, N’-tetramethyl-p-phenylenediamine], **Asc** [ascorbate], **Leak-I** (state 2 respiration through CI in the absence of ADP), **OXP-I** (state 3 respiration), **OXP-I, II** (parallel electron transport from CI and CII), **Leak-I, II** (leak respiration flux rate through CI and CII), **ETS** (maximal flux of the electron transport system), **CCO** (activity of CIV, cytochrome c-oxidase) and (B) pyruvate affinity at 20°C (see methods section for details).

Heart fibres from control and experimental fish were added to 2 mL chambers containing equilibrated Fish-MiRO5 in Oroboros Oxygraph-2k™ respirometers (Oroboros Instruments, Innsbruck, Austria). Oxygen was added into the gas phase above media prior to closing chambers to supersaturate Fish-MiRO5. Oxygen was maintained above 280 nmol. mL^−1^ throughout assays to maximize flux. Respiration was measured as the weight-specific oxygen flux [pmol O_2_ (mg wet weight · sec) ^−1^] following a titration-inhibition protocol outlined below (figure 2). The respiratory flux was calculated in real time as the negative time derivative of the oxygen concentration using Oroboros DatLab Software V 4.1.1.84 (Oroboros Instruments, Innsbruck, Austria).

The substrate-uncoupler-inhibitor titration protocol [63]–[65] tested mitochondrial function across the OXP system and ETS (figure 2). Complex I (CI) substrates (2 mM malate, and 10 mM pyruvate) were added to measure state II respiration through CI in the absence of ADP (denoted “Leak-I”). Excess ADP (2.5 mM) stimulated oxidative phosphorylation (OXP-I, state III respiration), and glutamate (10 mM) was added to saturate CI. Cytochrome *c* (Cyt *c*, 10 µM) was added to test outer membrane integrity. An increase in rate following exogenous Cyt *c* addition indicates outer mitochondrial membrane damage due to the loss of endogenous Cyt *c*. Phosphorylating respiration with CI and CII substrates (OXP-I, II) was measured by the addition of succinate (10 mM). NADH (0.5 mM) was then added to assess inner mitochondrial membrane damage. Leak respiration rates were also measured on combined CI and CII substrates by addition of atractyloside (750 µM, Leak-I, II) followed with repeated titrations of carbonyl cyanide *p*-(trifluoromethoxy)phenyl-hydrazone (FCCP, 0.5 µM) to uncouple mitochondria (denoted “ETS”). By the addition of rotenone (0.5 µM), malonate (15 mM) and antimycin a (1 µM), CI, II and III activities were inhibited respectively. Finally, the activity of cytochrome *c*-oxidase (CCO) was measured by the addition of the electron donor couple *N*,*N*,*N*′,*N*′-tetramethyl-*p*-phenylenediamine (TMPD, 0.5 mM) and ascorbate (2 mM) (figure. 2). Chemical background assays were run to account for the auto-oxidation of TMPD and ascorbate at the seven experimental temperatures and subtracted from CCO flux rates.

#### Reactive oxygen species detection

We used purpose built fluorimeters that were similar to those used by Hickey et al. [66] that consisted of LEDs with a peak excitation of 520 nm. The sensors were attached to Oroboros O2K oxygraph systems permitting the simultaneous measurement of ROS production and mitochondrial respiration rates. To calibrate the fluorimeter, 400 *p*mol of resorufin (25 µM) was added to each chamber prior to each assay. Horse-radish peroxidase (HRP, 2.5 U. mL^−1^) was added to complete the Amplex-Ultrared reaction. Superoxide dismutase (SOD, 24 U. mL^−1^) was then added to capture mitochondrial produced super-oxide and convert this to hydrogen peroxide (H_2_O_2_). Steady state rates were followed using DATLAB 4.3 and corrected for tissue mass and background activities.

#### Experiment 2: Determining the cardiac mitochondrial failure temperature

In these experiments, respiration and ROS were measured in heart fibres from naive fish at seven temperatures (*N = 8* per temperature) to elucidate the temperature when mitochondrial dysfunction (T_mt_) occurs and its relation to T_HF_ (figure 1). Respiration was measured at 15°C (average ocean temperature in winter), 17.5°C (tank acclimation temperatures), 20°C (average ocean temperature in summer), 25°C (maximal summer temperature), 27.5°C (T_HF_), 30°C and an extreme maximum of 32.5°C.

#### Experiment 3: Determining substrate affinity (Apparent Km) with increasing temperature

We tested the capacity (pseudo-affinity, or apparent K_m_; K_m app_) of mitochondria within permeabilized fibres to take up pyruvate or glutamate in the presence of malate (5 mM) and ADP (2.5 mM) at increasing *in situ* temperatures (20°C, 25°C, 27.5°C, 30°C and 32.5°C). The affinity for succinate was not tested as this is a derivative of acetyl-CoA and therefore, is dependent on pyruvate *in vivo*. Initial trials optimized pyruvate or glutamate concentrations at different temperatures. Based on these data, the respective substrate was titrated by stepwise substrate additions using microinjection pumps (Oroboros Tip O2K) until respiration flux appeared to be saturated (figure 2B). Michaelis-Menten curves were generated, and substrate-saturation curve kinetics were applied to determine K_m app_ and V_max_ values using nonlinear regression (Sigma Plot 12.0, San Jose, CA).

#### Experiment 4: ATP production with increasing temperature

The production of ATP was determined by following the changes in free extra-mitochondrial [Mg^2+^] indicated by a Mg^2+^-sensitive fluorescent indicator, Magnesium Green (MgG) [67]. We adapted this method to the Oroboros O2K oxygraph using a 503 nm LED for excitation and a 530 nm filter for emission. Experiments with permeabilized fish heart fibres were performed at 20°C, 25°C, 27.5°C, 30°C and 32.5°C. Fibres were added to Fish-MiRO5 in the oxygraph chambers with blebistatin (a myosin heavy chain inhibitor, 60 µM) and oubain (Na^+^-K^+^-ATPase inhibitor, 50 µM). MgG (5 µM) was then added and chambers were oxygenated. Leak-I was determined by the addition of malate (2 mM), pyruvate (5 mM) and glutamate (10 mM). Mg^2+^ free ADP (5 mM) was then added in excess to saturate mitochondria and obtain maximum ATP production via CI. Succinate (10 mM) was added to obtain ATP production by CI and CII. All complexes were then poisoned by the addition of rotenone (0.5 µM), malonate (15 mM) and antimycin a (1 µM). After the addition of each substrate or inhibitor, the assay was recorded for 5 minutes to obtain a clear signal.

To calibrate the ATP signal, separate titrations were performed to test the linearity and response of the MgG to sequential additions of Mg^2+^ free ADP and in separate titrations to fresh Mg^2+^ free ATP. ADP and ATP bound Mg^2+^ quenched MgG fluorescence linearly within the range the assays were conducted under. As expected, ATP which has a greater affinity for MgG quenched the MgG signal more for an equimolar amount of ADP. Using these data we derived ratios for each temperature of ATP fluorescence relative to ADP fluorescence. The same amount of ADP (10 µM) was added at each temperature and this resulted in a consistent fluorescence. This signal was used to determine the amount of ATP produced by multiplying the ADP signal by the appropriate ratio and this was then used as a re-calibration value.

### Calculations and Statistical Analyses

Heart rate was determined using a script written for Octave®, a numerical computational freeware. The Doppler output signal in Audacity® was converted to WAV format to use with Octave® which calculates the peak frequency heart rate. The signal envelope was first computed using a root mean square approach, and peaks were defined as any part of the signal greater than 50% of the maximum value of the envelope function. Sonogram data were analyzed using a repeated measures ANOVA followed by a post hoc Tukey test with a significance level of *P*≤0.05. In experimental fish, ABT was determined by segmented linear regression using the SegReg program (www.waterlog.info). R^2^ was computed from the sum of the squares of the distances of the points from the best-fit line determined by nonlinear regression using Prism5®. The plasma metabolite levels were determined from base peak height as detected by the GC and normalized to an internal standard (d4-alanine). Metabolites in the plasma samples were identified using an in-house methyl chloroformatate MS library of derivatized metabolites. This library contains MS spectra obtained from ultra-pure standards with the mass spectra saved and analyzed with AMDIS 2.65 software (www.amdis.net). A comparative metabolite profile was constructed and analyzed using R-software [68].

Respiratory control ratios (RCRs) were calculated as OXP-I/Leak-I, and uncoupled control ratios (UCRs) were calculated as ETS/OXP-I, II. A dose-dependent analysis conducted with Prism5® was used to individually test the temperature breakpoint where RCR and UCR change with heat stress. Increasing assay temperature was considered as an inhibitor and the RCR or UCR were considered as the response to the inhibitor. To test if outer mitochondrial membrane damage had occurred, the fractional increase in oxygen flux after Cyt *c* addition (Cyt *c*/OXP-I - 1) was calculated. The inner mitochondrial membrane damage (NADH/OXP-I, II - 1) was similarly tested. For both calculations a one-sample t-test was employed to test if measures differed from zero. OXP-I, II/Leak-I, II defined as RCR 2 [47] was also used as a proxy for measuring inner membrane permeability [64]. Control or limitation by the ETS was determined by the comparison of leak respiration by atractyloside inhibition relative to uncoupled respiration (Flux control ratio FCR; Leak-I, II/ETS) [69]. In mitochondrial respiration assays, differences across temperatures and between control and experimental fish were evaluated with a one or two-factor ANOVA as appropriate, followed by a post hoc test (Tukey). Dose dependent analysis with Prism5® was also used to test if ROS production changed with heat stress. In this analysis, a log(agonist) vs. response curve was used where increasing assay temperature was the agonist while ROS production was the response. All statistical tests were run using SigmaPlot® version 12 (Systat Software, Inc., San Jose, California) unless otherwise stated. Data were reported as means ± SEM (*N* is the number of fish) unless otherwise stated.

## Results

### Thermal Tolerance Limits of Cardiac Function

**Figure 3:**
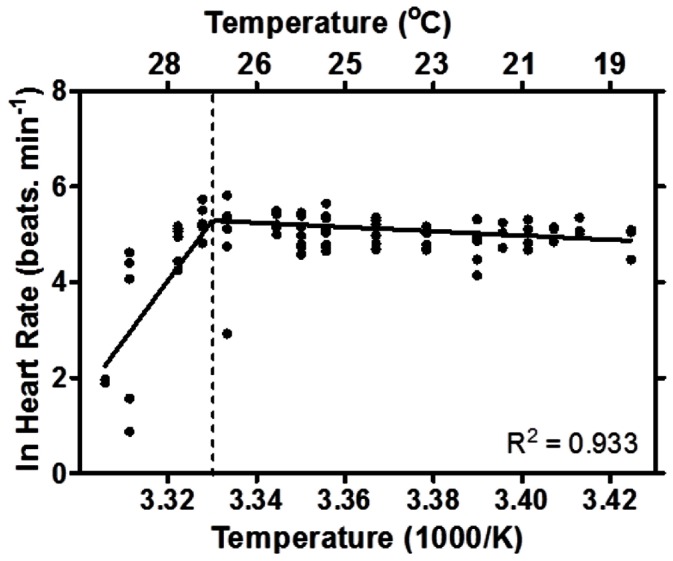
Arrhenius break temperature (ABT) of heart rates of single individuals of *N. celidotus*. The ABT was 27.81±0.39°C and values were expressed as individual heart rates per temperature (*N = 8*).

**Figure 4:**
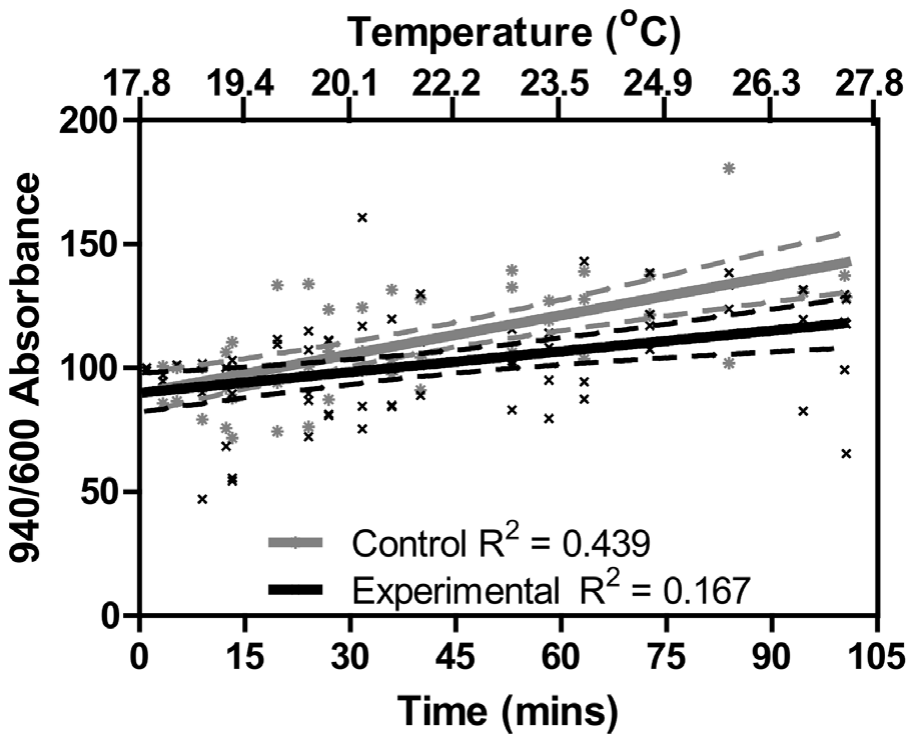
Changes in haemoglobin oxygen saturation in *N. celidotus* expressed as the change of infrared (940 nm) to red (600 nm) absorbance ratio in control (*N* = 4, grey x) and experimental (*N = 6,* black x) fish. Values are expressed for individual fish and linear regression was fitted for control (grey line) and experimental (black line) data with 95% CI in daggered lines. Control fish did not experience changes in temperature; therefore regressions were performed on absorbance ratios relative to time (top x-axis). Regression analysis for experimental fish were performed on absorbance ratios relative to temperature (bottom x-axis). Goodness of fit is given as R^2^.

With increasing temperature *N. celidotus* maintained heart rate until an average temperature of 27.8±0.4°C (R^2^ = 0.93) (figure 3). This indicates the critical temperature of heart failure (T_HF_) occurs above 27.5°C (figure 3). The heart rate at the beginning of the experiment for the control fish did not change compared to heart rate at the end of the experiment when the experimental fish had attained T_HF_ (*N* = 8, data not shown). Haemoglobin oxygen saturation of experimental fish did not differ from control fish (*p*≥0.05; figure 4). The percentage change in the 940∶600 ratio remained relatively constant in control and experimental animals as temperature gradually increased to T_HF,_ and 95% confidence intervals (95% CI) for linear regression overlapped (figure 4).

Metabolite analysis of plasma from the cardiac function experiment showed that the only glycolytic intermediate detected was lactate (table 1). Lactate levels significantly increased by 1.55-fold in experimental plasma, compared to controls. Five TCA cycle intermediates were identified in plasma; citrate, cis-aconitate, α-ketoglutarate, succinate and malate. Citrate, succinate, and malate were consistently detectable in both control and experimental plasma samples (*N = 6,* table 1). Although citrate appeared to increase in acutely heat stressed plasma, only succinate was significantly elevated and malate was significantly lowered. More essential amino acids (EAAs) were significantly elevated in heat stressed plasma (five amino acids) compared to non-essential amino acids (NEAAs; three amino acids) (table 1). Similar to measures in plasma, lactate trended higher (33%) in cardiac tissue of acutely heat stressed fish (*p = *0.07; table 2). The activity of LDH significantly increased by 50%, indicating that heat stressed hearts had up-regulated anaerobic capacities during temperature exposure time while CS and G6PDH remained unchanged with heat exposure.

**Table 1:** Patterns of metabolites (glycolytic, TCA cycle intermediates, amino acids) in control and experimental fish plasma (*N = 6)*.

Metabolite		Increase/Decrease in Experimental plasma	Experimental/Control fold change	
**Glycolytic Intermediates**				
Lactate		↑		1.55*
**TCA Cycle Intermediates**				
Citrate		↑		1.55
Succinate		↑		1.35*
Malate		↓		0.92*
**Essential Amino Acids (EAAs)**				
Leucine	↑			2.38*
Lysine	↑			2.80*
Phenylalanine	↑			2.02*
Tryptophan	↑			2.29*
Valine	↑			2.46*
**Non-essential Amino Acids (NEAAs)**				
Cysteine	↑			1.84*
Glutamate	↑			1.66*
Tyrosine	↑			2.24*

Arrows in column B indicate the increase or decrease in accumulation of metabolite in experimental plasma compared to control plasma.

* denotes significant change in metabolite at *p*≤0.05 in experimental plasma compared to control plasma (column C).

**Table 2:** Glycolytic intermediates and enzymes in control and experimental fish cardiac tissue.

	Control	Experimental
**Glycolytic Intermediates**		
Lactate	106.61±18.46	142.39±10.44 (*p* = 0.07)
**Enzymes**		
G6PDH	369.59±76.19	388.83±26.86
Citrate Synthase	198.06±80.19	176.35±34.83
Lactate Dehydrogenase	1050.11±92.75	1682.83±168.00*

Unit for lactate and enzymes is µmol. min^−1^. mg protein^−1^.

Values are means ± S.E.M (*N* = 4*).*

* denotes significant change at *p*≤0.05.

### Mitochondrial Bioenergetics

#### Mitochondrial function in heart fibres from fish acutely exposed to heat stress

**Figure 5:**
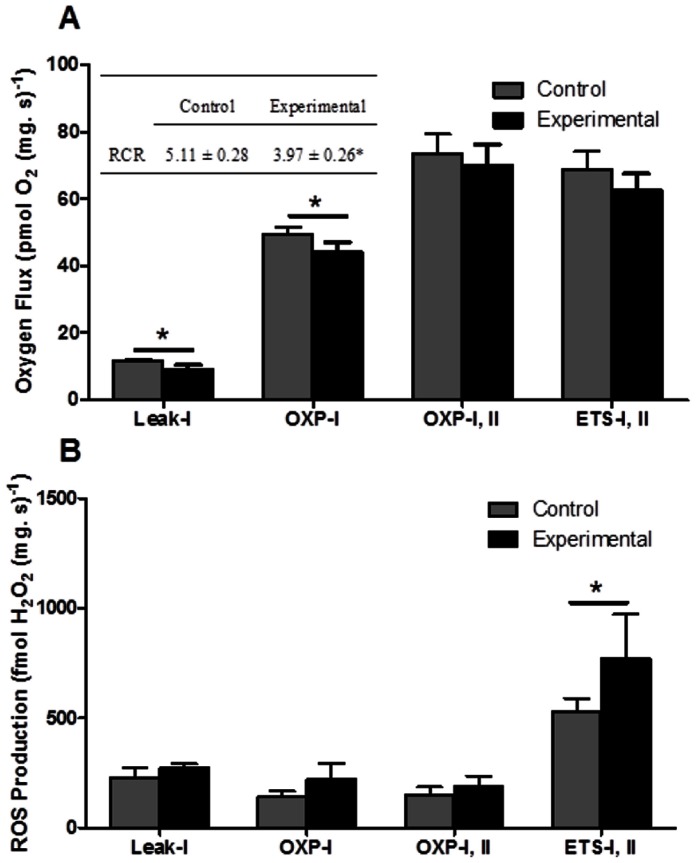
In permeabilized heart fibres from control (grey bars) and experimental (black bars) fish (*N* = 8) (A) mean respirational flux (insert) RCR values for control vs experimental mitochondrial respiration and (B) ROS production. See methods for mitochondrial respiration state details. Values are means ± S.E.M. The asterisks denote a significant difference between control and experimental fish at a given respiration state at *p*≤0.05.

Following an acute exposure to increasing temperature permeabilized heart fibres showed few differences from control fish (figure 5A). However, experimental fish showed significantly lower Leak-I and OXP-I fluxes, compared to control fish. This significantly depressed the RCR by ∼22% with temperature exposure (*p<*0.05; figure 5A insert). The OXP flux fuelled by CI and CII substrates and uncoupled rates (ETS) between control and experimental fish remained unaltered (figure 5A). ROS production was significantly elevated when mitochondria were uncoupled, although acute heat stress did not change ROS production in Leak-I, OXP-I or OXP-I, II states (figure 5B).

#### Impacts of *in situ* heat stress on permeabilized heart mitochondria

**Figure 6:**
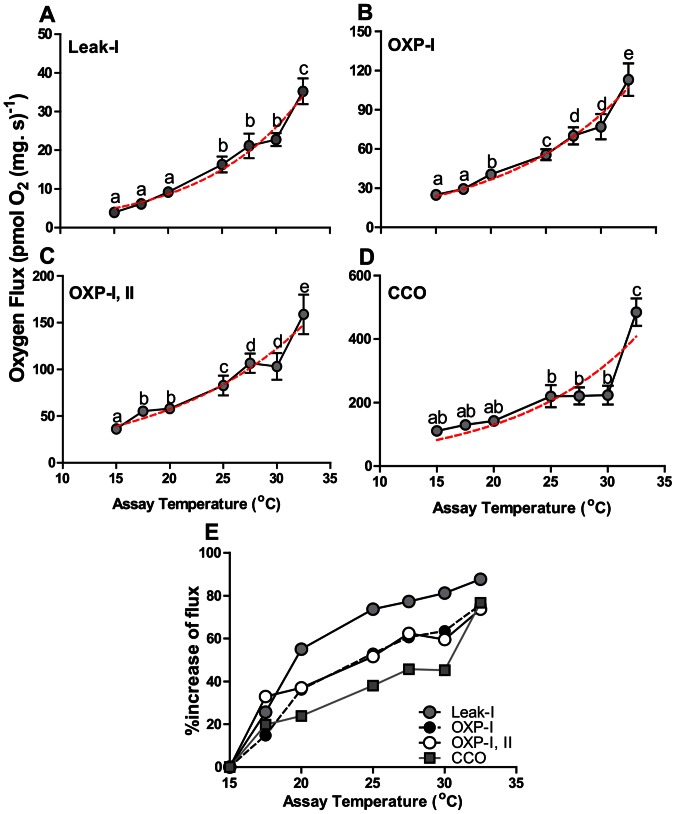
Cardiac mitochondrial respirational flux measured at increasing assay temperature in permeabilized cardiac fibres of *N. celidotus* (*N* = 8 per assay temperature). (A) Leak-I respiration with CI substrates malate and pyruvate; (B) OXP-I respiration with CI substrates malate, pyruvate and glutamate; (C) OXP-I, II respiration with CI and CII substrates; (D) CCO respiration of complex IV; (E) percentage increase of respiration from initial rates at 15 oC for Leak-I (grey circles), OXP-I (black circles), OXP-I, II (white circles) and CCO (grey squares). Values are means ± S.E.M. Points with similar letters are not significantly different at *p≤*0.05. One-site saturation exponential curves (red dotted lines) were fitted for each graph (A-D) and goodness of fit is given as R^2^.

All components (Leak-I, OXP-I, OXP-I, II, CCO respiration, RCR, UCR) of mitochondrial phosphorylation were sensitive to increased temperatures (figure 6, 7A). An increase in temperature showed the expected exponential increase in Leak-I, OXP-I, OXP-I, II and CCO respiration. However, the goodness of fit of the exponential curve for OXP-I, II (R^2^ = 0.58, figure 6C) and CCO (R^2^ = 0.53, figure 6D) were low. This likely resulted from lower than expected flux rates at 30°C, which fell below predicted values when an exponential curve was fitted (figure 6C, D). The thermal mediated increase in flux appeared to plateau between 27.5 and 30°C, and this was most evident in OXP-I, II and CCO states (figure 6C, D).

**Figure 7:**
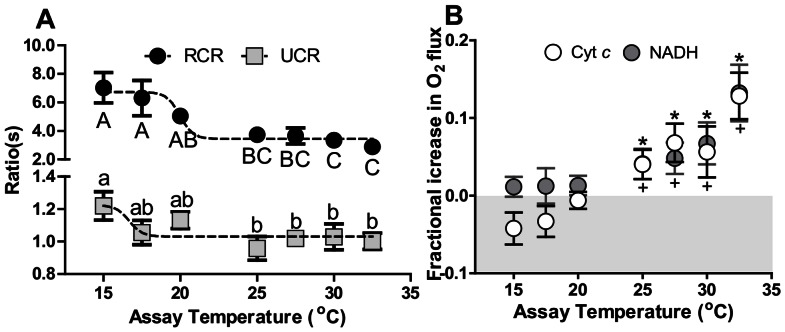
Components of cardiac mitochondrial respirational flux measured at increasing assay temperature in permeabilized cardiac fibres of *N. celidotus*. (A) Mean RCR (black circles, OXP-I/Leak-I, *N* = 8 per assay temperature) values and mean UCR (grey squares, ETS/Oxp-I, II, *N* = 8 per assay temperature) values. A dose-dependent analysis curve was fitted to both RCR and UCR data (dashed line) (B) Mean fractional increase in OXP-I respiration with Cyt *c* addition (white circles) and mean fractional increase of OXP-I, II respiration with NADH addition (grey circles). The asterisks denote the increase in respiration after Cyt *c* addition is significantly different from zero. The plus sign denotes increase in respiration after NADH addition is significantly different from zero at *p*≤0.05.

Leak-I, OXP-I, OXP-I, II and CCO rates were expressed as a percentage increase from those at 15°C in order to illustrate the relative effects of increasing temperature for each state. Overall, a more substantial increase was observed for Leak-I rates, compared to other measured states (grey circles, figure 6E). At 25°C Leak-I rates had increased by 74%, whereas other mitochondrial states increased by only 50% or less. The OXP-I and OXP-I, II rates had initial increases of 33% at 17.5°C. At temperatures above 17.5°C, the percentage increase in flux rates were only marginal (∼15%, figure 6E). The percentage increase of CCO appeared to be the most constrained below 30°C, as the percentage increase in rates remained under 40% (grey squares). At 32.5°C, OXP-I, OXP-I, II and CCO had increased by ∼75% (figure 6E). This was further reflected in the ratios of CCO/OXP-I, II and CCO/ETS where values at 32.5°C were significantly higher than all temperatures measured except at 15°C (table 3).

**Table 3:** Ratios based on mitochondrial respirational flux in *N. celidotus* at 15°C, 17.5°C, 20°C, 25°C, 27.5°C, 30°C and 32.5°C. OXP-I, II/Leak-I, II (also termed RCR 2, [50]) ratio is a simple proxy of inner membrane permeability.

State	15°C	17.5°C	20°C	25°C	27.5°C	30°C	32.5°C
OXP-I, II/Leak-I, II (RCR 2)	2.22±0.19^a^	2.28±0.11^a^	2.33±0.15^a^	2.06±0.19^ab^	2.04±0.24^ab^	1.77±0.17^ab^	1.55±0.11^b^
Leak-I, II/ETS (FCR)	0.38±0.04^a^	0.43±0.04^a^	0.40±0.03^a^	0.49±0.04^ab^	0.55±0.04^bc^	0.56±0.04^bc^	0.63±0.05^c^
CCO/OXP-I, II	2.96±0.35^ab^	2.37±0.20^bc^	2.45±0.21^bc^	2.96±0.35^bc^	2.45±0.23^c^	2.13±0.10^c^	3.26±0.36^a^
CCO/ETS	2.50±0.34^ab^	2.29±0.11^b^	2.15±0.22^b^	2.37±0.27^b^	2.08±0.09^b^	1.94±0.12^b^	3.24±0.46^a^

The Leak-I, II/ETS (termed flux control ratio, FCR) provides a measure of ETS capacity relative to the leak respiration state when phosphorylation is inhibited by atractyloside. CCO/OXP-I, II and CCO/ETS are measures of the capacity of cytochrome *c* oxidase (CCO) relative to maximum phosphorylation (OXP-I, II) or the ETS respectively.

Values are means ± S.E.M. (*N* = 8 at each temperature).

Means with the same letter of the same case are not significantly different from one another *(p≤*0.05).

**Figure 8:**
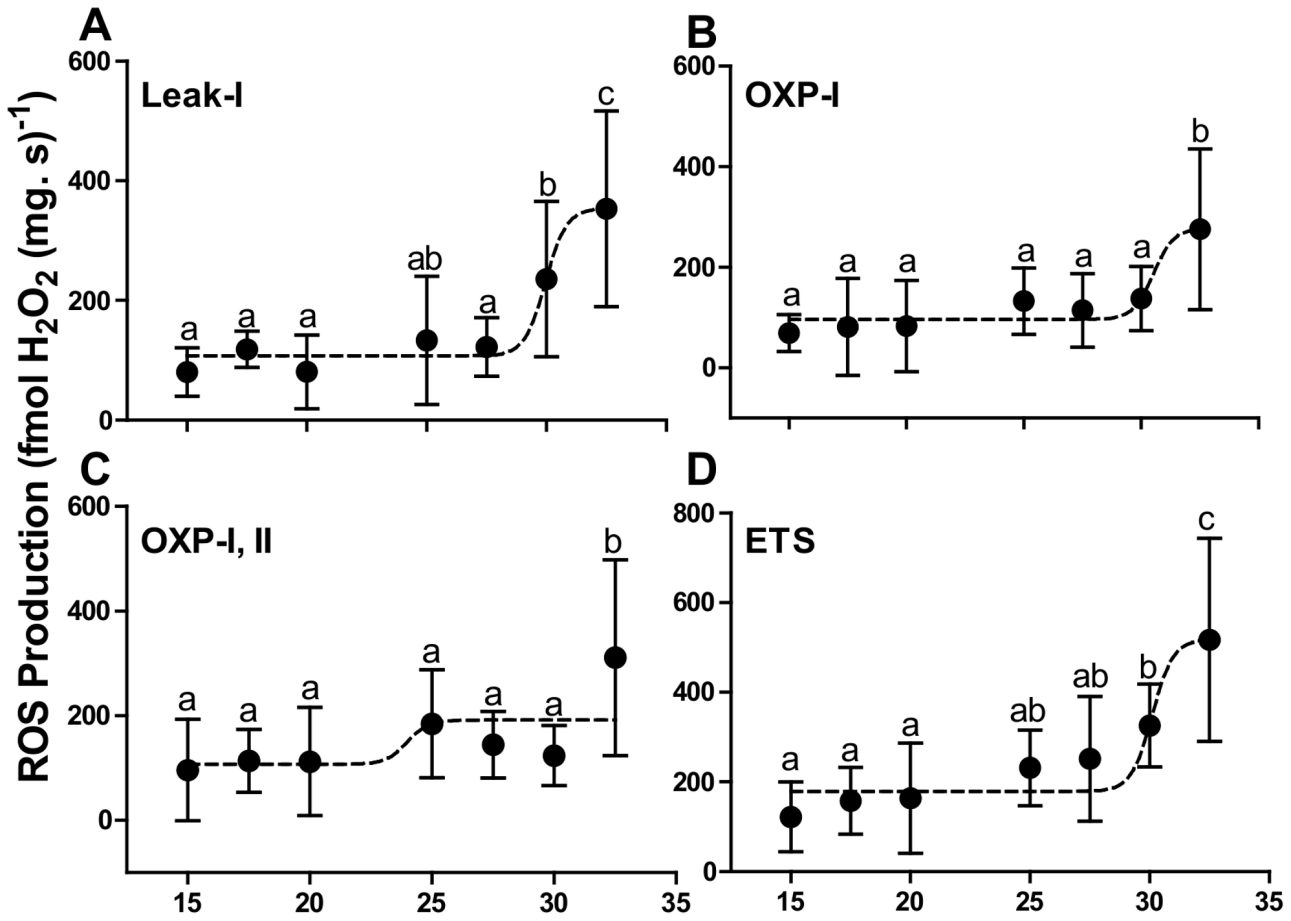
ROS production by permeabilized heart fibres in (fmol H_2_O_2_ (mg. s) ^−1^) with increasing temperature. (A) ROS production in the Leak-I state; (B) ROS production in the OXP-I state; (C) ROS production in the OXP-I, II state; (D) ROS production in the ETS uncoupled state. A dose-dependent agonist analysis curves were fitted for all states (black daggered lines). Values are means ± S.E.M for *N* = 8. Means sharing the same letter are not significantly different from one another at *p*≤0.05.

RCR values remained above 4 at assay temperatures 20°C and lower, indicating that mitochondria were robust and not damaged [31]. A dose-dependent curve showed a breakpoint at ∼20°C (19.97±0.42°C), indicating a depression in RCR values after this temperature. From 25°C upwards, RCRs were significantly depressed suggesting that above this temperature OXP capacity with CI substrates is compromised (figure 7A black circles). The RCR was depressed to 3.34±0.29 at 30°C which was similar to values at 32.5°C (figure 7A). The OXP-I, II/Leak-I, II ratio (RCR-2) displayed a similar trend to RCR as it was significantly depressed at and above 25°C (table 3). The UCR (ETS/OXP-I, II) decreased above 20°C. Furthermore, when increasing assay temperature was considered as an inhibitor and a dose-dependent analysis curve was fitted to UCR values, a breakpoint was found around 17.5°C (16.68±1.49°C, figure 7A grey circles). Comparison of the flux control ratio (FCR, ETS/Leak-I, II) further indicated that the ETS may have become limiting above 20°C (*p≤0.05,* table 3). The fractional increase in oxygen flux resulting from Cyt *c* addition significantly increased OXP-I rates from 20°C, indicating outer mitochondrial membrane damage (figure 7B). While the inner membrane is normally impermeable to NADH, the addition of NADH increased OXP-I, II respiration above 20°C demonstrating damage to the inner mitochondrial membrane (grey circles, figure 7B). Following an increase in temperature *in situ*, there was a gradual increase in ROS production in the Leak-I and uncoupled states (figure 8A, D). Dose-dependent analysis determined breakpoints after 30°C for Leak-I (29.96±0.31°C), OXP-I (30.53±0.53°C) and ETS (30.12±0.33°C) states indicating ROS production increased after this temperature (figure 8A–B, D). In the OXP-I, II state the breakpoint was found at 23.99±3.26°C however, ROS production in this state was significantly elevated only at 32.5°C (*p≤0.05*) (figure 8C).

#### Apparent substrate kinetics of heat stressed heart fibres

**Figure 9:**
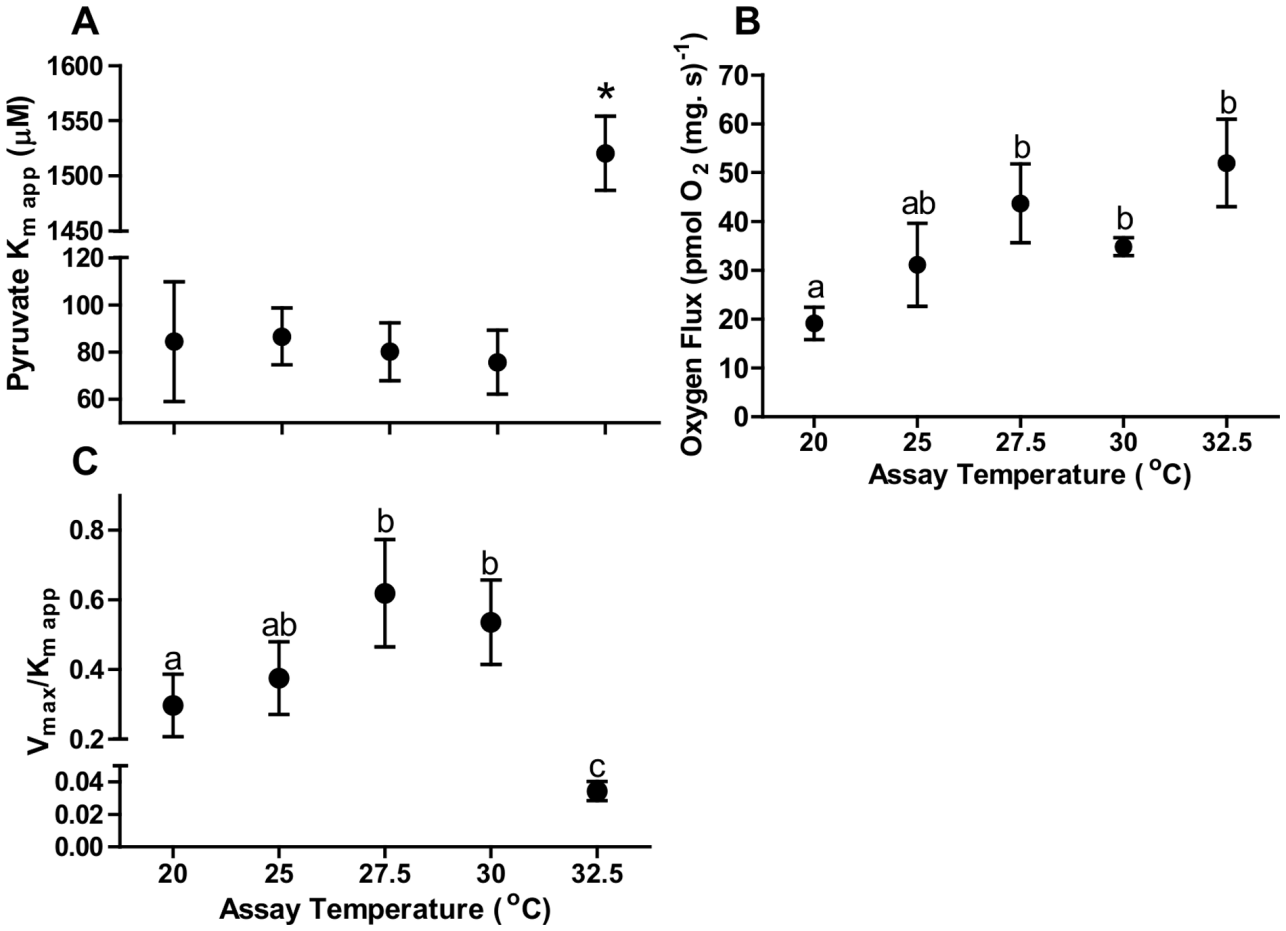
Pyruvate affinity in cardiac fibres of *N. celidotus* examined at 20°C, 25°C, 27.5°C, 30°C and 32.5°C. (A) Pyruvate concentration giving half maximal respiration rate (K_m app_); (B) maximal pyruvate stimulated respiratory flux rate (V_max_) (C) ratio of V_max_/K_m app_ as an indicator of substrate efficiency. Values are mean s± S.E.M for *N* = 8. Means sharing the same letter are not significantly different from one another at *p*≤0.05.

In the presence of malate and excess ADP the stepwise addition of pyruvate at all temperatures accelerated the oxygen consumption rate of heart fibres (figure 2B). The apparent affinity of heart fibres for pyruvate was similar at all temperatures until 32.5°C when the K_m app_ for pyruvate increased approximately 20-fold higher than all other temperatures measured (figure 9A). The predicted V_max_ extrapolated from Michaelis–Menten curves indicated a significant increase with increasing temperatures and plateaued at 27.5°C (figure 9B). When the mitochondrial V_max_/K_m app_ ratio for pyruvate (analogous to the k_cat_/K_m_ measure of enzymatic efficiency) was compared between temperatures, increasing temperatures led to a significant increase in the ratio until 30°C (figure 9C). At 32.5°C this ratio decreased 14-fold indicating a substantial loss of kinetic efficiency. The affinity for glutamate to initiate CI respiration remained unchanged at all temperatures, and the K_m app_ of mitochondria within fibres was in the mM range (data not shown).

#### Relative ATP production

**Figure 10:**
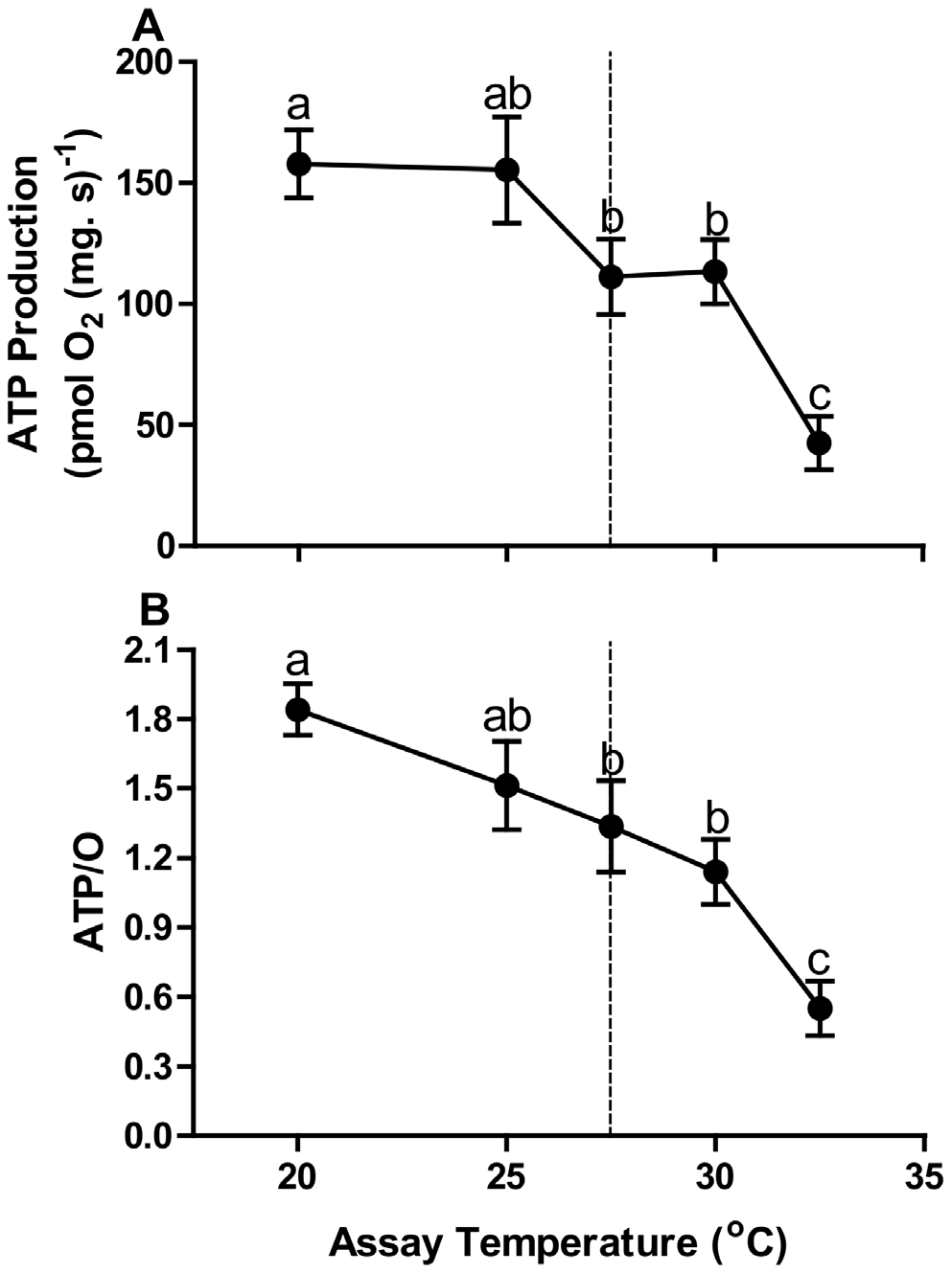
The production of ATP across increasing assay temperatures at 20°C, 25°C, 27.5°C, 30°C and 32.5°C in permeabilized *N. celidotus* cardiac fibres; (A) at maximal respiration OXP-I, II and, (B) the ratio of ATP production and OXP-I, II respiration. A dashed line at 27.5°C indicates T_HF_. Values are means ± S.E.M for *N* = 6. Means sharing the same letter are not significantly different from one another at *p*≤0.05.

The production of ATP in permeabilized fibres with increasing assay temperature supported mitochondrial phosphorylation trends we identified in this study (figures 6–9). ATP production was measured in OXP-I, II and by 25°C it was lower than at 20°C and was severely inhibited by 32.5°C (figure 10A). When expressed as a ratio of ATP production to oxygen consumption in the OXP-I, II state, the ATP/O ratio was significantly depressed at 25°C; and ATP/O ratios were depressed by 3.5-fold at 32.5°C relative to values at 20°C (figure 10B).

## Discussion

The contributing role of mitochondria to HF has largely been dismissed because oxygen flux through mitochondria at T_max_ appeared unhindered in most ectotherms studied to date. However, this paradigm has been developed from mechanistic examinations concerned less with mitochondrial efficiencies, integrities and their significant roles in apoptosis [11], [35], [70]. In contrast, this study provides comprehensive evidence that mitochondrial function is impaired concurrent with or prior to HF in a temperate fish species. In the presence of multiple and non-limiting substrate concentrations to maximize respiration flux, we showed that several mitochondrial components are compromised before the onset of T_HF_ (27.5°C). Specifically, both OXP efficiency and the absolute production of ATP are impaired prior to T_HF_. The association of these decreases coincides with succinate accumulation in heat stressed hearts despite excess oxygen (*in vitro*) and no loss of haemoglobin oxygen saturation (*in vivo*). These data suggest that impairment of oxygen consumption by heart mitochondria precedes oxygen limitation. While mitochondrial respiration rates further increased at supra-physiological temperatures, this depended on substrate concentrations that are not biologically relevant. Importantly, the loss of Cyt *c* and aerobic generation of ATP production should drive apoptosis or promote necrosis. These significant findings provide insight into current descriptions of analogous challenges in mammals; notably mitochondrial induced cytopathic hypoxia. This condition occurs in haemorrhagic shock, hyper-pyrexia and sepsis [71], [72]. In these pathological settings, even with sufficient blood oxygenation, mitochondrial respiration is impaired.

### The Limits on Heart Function by Acute Heat Stress

The heart is considered to be the most temperature sensitive organ in animals with circulatory systems [17], [73], and according to the Fick principle cardiac output (CO, the product of heart rate and stroke volume) increases in conditions of high oxygen demand such as heat stress [14]. Demonstrably, cardiac function of *N. celidotus* was altered during exposure to increasing temperatures (figure 3). Given that there was a limited increase in beat rate, changes were most likely augmented by alterations in stroke volume which is common for fishes (figure 3) [73]. The change in cardiac function was not a response to blood oxygen levels as haemoglobin saturation *in vivo* did not decrease with acute heat stress (figure 4). This is of great interest because the primary cause of thermally-mediated HF in ectotherms has been generally subscribed to the decrease in oxygen solubility with temperature [2], [12], [13], [74]. Such a decrease in blood oxygen content should lower mitochondrial oxygen supplies and limit OXP. Numerous studies have identified that the solubility of oxygen in water decreases with increasing environmental temperatures [2], [12], [13], [75]–[77]. However, oxygen diffusion rates increase in warm water [14], [15], and we found no immediate decline in blood saturation that can be associated with hypoxia.

In general, glycogen and free glucose become the main metabolic fuels in stressed and hard-working vertebrate muscles including the heart. Increased plasma lactate and succinate, and decreased malate of thermally challenged *N. celidotus* indicate enhanced anaerobic metabolism, interrupted ETS, or mitochondrial disruption in this species (table 1). This conclusion is supported by an increase in LDH activities in heart tissues from fish exposed to acute heat stress (table 2). Elevated glycolytic capacities may represent attempts to offset increased ATP demands under heat stress [78], and compensatory energetic responses to loss of ATP production have been observed in other studies on heat stressed ectotherms [79]. Furthermore, the accumulation of EAAs in experimental plasma could be attributable to increased protein catabolism to fuel metabolism in the presence of heat stress [80]. However, the accumulation of plasma glutamate, a NEAA that is a *TCA* cycle intermediate and neurotransmitter, may also be associated with a global decrease in mitochondrial oxidation with acute heat stress (table 1). Thus the altered patterns of metabolites in this study imply the onset of mitochondrial dysfunction for this species (table 1, 2).

### Thermal Sensitivity of Cardiac Mitochondria

#### Mitochondrial function following acute heat stress in intact fish

Following acute heat stress exposure, *N. celidotus* hearts most likely face decreased OXP efficiencies supported by lower Leak-I and OXP-I fluxes in experimental animals, compared to controls (figure 5A). These changes were reflected by decreased RCRs in acutely temperature exposed fish (figure 5A insert). This can be caused by irreversible changes in mitochondrial inner membrane integrity with extreme heat stress which has been observed in mammalian heart fibres, and this increases the permeability to ions, decreases membrane potential, and results in a decreased OXP efficiency [31], [70]. The depression in RCR and relative increase in Leak-I may indicate a response to ROS as production trended higher in heat stressed fibres in OXP states and was significantly higher in the chemically uncoupled ETS state (figure 5B). Mitochondrial uncoupling at high temperatures is thought to result from increased superoxide up-regulating uncoupling proteins [81]. However, the significantly higher production of ROS in the experimentally exposed heart fibres could also be attributed to damage at either CI or CIII (figure 5B) as these are the most commonly accepted sites of mitochondrial ROS production [81].

#### Impacts of increasing *in situ* temperature on heart mitochondria

Overall, substantial changes to both the inner and outer mitochondrial membranes were evident at 25°C before T_HF_. Leak-I rates increased by 75% at 25°C relative to those at 15°C (figure 6E) indicating increased inner membrane permeability. Whereas OXP states were less temperature sensitive and this resulted in a measurable depression of the RCR (figure 7A) suggesting increased oxygen flux rates are required to maintain mitochondrial membrane potentials. An initial dose-dependent analysis indicated that RCR values were inhibited by 20°C when increasing temperature was considered as an inhibitory dose. A further robust ANOVA analysis proved that this depression had started by 25°C (figure 7A, black circles). These depressed RCR indices should decrease phosphorylation efficiencies and capacities [47], [82], [83]. Moreover, the addition of NADH increased OXP-I, II flux prior to T_HF_ indicating increased permeability of the inner membrane to this otherwise impermeable molecule (figure 7B).

The integrity of the outer mitochondrial membrane was also compromised above 20°C as respiration following cytochrome *c* addition increased before T_HF_ (figure 7B). The loss of outer mitochondrial permeability leads to the release and depletion of Cyt *c* from mitochondria [29], [84]. A substantial loss of Cyt *c* clearly depresses OXP flux which can potentially affect superoxide scavenging [85]. At 25°C this can prove problematic because ROS production increased as Leak-I respiration increased (figure 6A, 8A). A key producer of ROS is CI of the ETS and its involvement in cardiac failure has been previously studied [86]–[88]. Thus, despite an inferred decrease in membrane potential via increased Leak-I, ETS complexes remain reduced and can elevate ROS production (figure 7A, 8A).

OXP flux rates fuelled by CI and CII significantly increased up to an assay temperature of 27.5°C (T_HF_) and then plateaued until 30°C (figure 6C). The plateau between 27.5 and 30°C likely indicates a transition in cardiac mitochondrial function. A similar plateau was also apparent for CCO. Given that this assay tests a single component of the ETS, these data suggest the plateau observed in OXP-I, II may result from limits on CCO until 30°C. A sudden increase in OXP-I, II and CCO rates was seen above 30°C and this was responsible for the poor fits to exponential curves predicted by a standard Q_10_ relationship (figure 6D). CCO is assumed to be abundant or in excess in mitochondria because CCO flux capacity is in excess relative to that required for maximal OXP and ETS flux rates [40], [89], [90]. In our study CCO flux was variable relative to OXP-I, II, yet declined relative to ETS flux (CCO/ETS, table 3) as temperature increased until 32.5°C. Therefore, maximal ETS electron flux probably does not require support by the total catalytic capacity of CCO. CCO is however regulated *in vivo* by molecules such as NO, H_2_S, ATP and O_2_ [91]–[94], consequently the full CCO flux as determined *in vitro* may not be realised *in vivo*. By 30°C, CCO flux had only risen by 40% of the initial 15°C flux (figure 6D, E). This can be caused by a relative decrease in CCO (table 3) which impairs oxygen binding capacities *in vivo* [94], in particular in the presence of regulator molecules. Notably, a proportionate decrease in CCO relative to OXP also occurs in other ectotherms species with increasing temperature [46], [95]. In this study we measured CCO flux well above the T_HF_, and by 32.5°C, CCO flux had increased by 75% compared to values at 15°C (Figure 6E). This sudden rise in CCO flux suggests a transition in CCO function or a change in affinity for its substrates O_2_ or Cyt *c* [96].

Decreases in the mitochondrial ATP production and in the transfer of energy through the phosphor-transfer kinases contribute to HF in mammalian cardiac muscle [97]. This study directly tested ATP production from heart mitochondrial fibres with increasing temperature and found that ATP production decreased with increasing temperature (figure 10). At 25°C ATP production was lower than synthesis rates at 20°C, furthermore by 27.5°C, ATP production rates dropped by 28% compared to rates at 20°C (figure 10A). ATP demands will likely increase with rising temperatures due to increased demands on cardiac output and simple thermodynamic effects on ATPases in general. ATP/O values were already 18% lower at 25°C, compared to values at 20°C (figure 10B). This indicates that at 25°C less ATP is made by cardiac mitochondria and they require more oxygen to do so prior to T_HF_. These data are the first to directly show that the fish heart is limited by depressed mitochondrial ATP production as T_HF_ approaches.

#### Substrate utilization following heat stress

Pyruvate is an important substrate for heart mitochondrial oxidation and this appears to be the case for *N. celidotus* (figure 9). The *K*_m app_ for pyruvate can be set by properties of the pyruvate transporter or of pyruvate dehydrogenase (PDH) and in ectotherms has been mainly examined in skeletal muscle mitochondria [98]. In this study, the *K*_m app_ values for pyruvate in the 20–30°C range (∼81 µM, figure 9A) are higher than values for isolated trout red and white skeletal muscle mitochondria (46 and 37 µM, respectively) [99], [100], rat heart mitochondria (∼40 µM) [101], and considerably higher than values found in isolated carp red muscle mitochondria (<5 µM) [99]. The affinities of *N. celidotus* mitochondria for pyruvate remained constant between 20–30°C indicating flux rates will be maintained *in vivo* until 30°C (figure 9A). However, at 32.5°C *K*_m app_ values for pyruvate increased by ∼20-fold to 1.52±0.03 mM. Although this is similar to values obtained for goldfish muscle mitochondria (1.17 mM) [102], these concentrations are considerably higher than the physiological intracellular range for pyruvate concentrations across phyla [98].

A gradual increase in both the pyruvate *V*_max_ and the *V*_max_/*K*_m app_ ratio were observed in this study between 20–30°C (figure 9B, C). But *V*_max_/*K*_m app_ ratio was depressed by ∼14-fold at 32.5°C, compared to values at 20°C (figure 9C). Assuming that the *V*_max_/*K*_m app_ is analogous to the k_cat_/K_m_, 30°C would represent the upper limit of mitochondrial respiration *in vivo* for *N. celidotus.* The sudden change in the mitochondrial affinity for pyruvate at 32.5°C may again reflect a transitional change in the inner mitochondrial membrane (figure 9B) and perhaps is the same as that reflected in CCO flux rates (figure 6D). A more fluid inner mitochondrial membrane can impair pyruvate transporter function and therefore potentially decrease pyruvate affinities [103]. In addition, given that pyruvate is imported electrogenically a thermally mediated loss of membrane potential may impair respiration at low pyruvate concentrations. This is consistent with the drop in ATP synthesis capacity and RCR values at higher temperatures. Lastly, all previous work exploring mitochondrial function in heat stressed mitochondria used substrates such as pyruvate at saturating concentrations and many studies showed function well above T_HF_ [8], [104]. Our data indicate that in this species mitochondria most likely cannot work above 30°C as cytosolic concentrations of pyruvate will be too low.

#### Is the hot heart limited by mitochondria?

Biological systems are dependent on the efficiencies and stabilities of systems, and high respiration flux rates are futile if inadequate amounts of ATP are formed. Despite continued mitochondrial respiration at all states during exposure to temperatures far above the upper tolerance limit of *N. celiodotus*, the lack of coincident ATP production (figure 10) reveals that maximal rates of mitochondrial respiration can be misleading. Thus, previous studies that found ectotherm mitochondrial respiration to be robust well above species T_max_ do not preclude the possibility of a mitochondrial role in thermally induced HF [8], [104]. In fact, given 1) the depression in ATP production and RCRs and 2) patterns of Cyt *c* and NADH changes, we suggest that altered mitochondrial function and stability is a critical element in HF.

The deprivation of energy plays a major role in HF in the mammalian model. In this model metabolism of the heart is determined by substrate utilization, oxidative phosphorylation and ATP transfer/utilization of the mitochondria [105]. Inadequacy or a break down in any or all of these components will contribute to HF. Since substrate utilization by the mitochondria is affected after T_HF_ in *N. celidotus,* the cellular uptake of fuels to support oxidative phosphorylation is probably not disrupted enough to induce HF prior to T_HF_ (figure 9). In contrast, heat stress clearly depresses the production of ATP/energy via oxidative phosphorylation which is exacerbated by an increasingly leaky inner mitochondrial membrane and decreased uncoupling capacity (figure 6, 7, 10). Notably all mitochondrial components were studied at maximum oxygen saturation. Therefore changes observed are a direct impact of elevated temperature stress. Heat stress can further limit contractile function of the heart due to inadequate ATP production leading to mechanical failure [105], but further investigation on ATP transfer/utilization of cardiac mitochondria is needed. Clarifying these specific mechanisms that lead to HF should provide a powerful biomarker for predicting the impacts of temperature change on marine biodiversity.
